# Protein structure prediction in the era of AI: Challenges and limitations when applying to *in silico* force spectroscopy

**DOI:** 10.3389/fbinf.2022.983306

**Published:** 2022-10-07

**Authors:** Priscila S. F. C. Gomes, Diego E. B. Gomes, Rafael C. Bernardi

**Affiliations:** Department of Physics, College of Sciences and Mathematics, Auburn University, Auburn, AL, United States

**Keywords:** artificial intelligence, protein folding, adhesins, molecular dynamics, force spectroscopy, *Staphylococcus* infection

## Abstract

Mechanoactive proteins are essential for a myriad of physiological and pathological processes. Guided by the advances in single-molecule force spectroscopy (SMFS), we have reached a molecular-level understanding of how mechanoactive proteins sense and respond to mechanical forces. However, even SMFS has its limitations, including the lack of detailed structural information during force-loading experiments. That is where molecular dynamics (MD) methods shine, bringing atomistic details with femtosecond time-resolution. However, MD heavily relies on the availability of high-resolution structural data, which is not available for most proteins. For instance, the Protein Data Bank currently has 192K structures deposited, against 231M protein sequences available on Uniprot. But many are betting that this gap might become much smaller soon. Over the past year, the AI-based AlphaFold created a buzz on the structural biology field by being able to predict near-native protein folds from their sequences. For some, AlphaFold is causing the merge of structural biology with bioinformatics. Here, using an *in silico* SMFS approach pioneered by our group, we investigate how reliable AlphaFold structure predictions are to investigate mechanical properties of *Staphylococcus* bacteria adhesins proteins. Our results show that AlphaFold produce extremally reliable protein folds, but in many cases is unable to predict high-resolution protein complexes accurately. Nonetheless, the results show that AlphaFold can revolutionize the investigation of these proteins, particularly by allowing high-throughput scanning of protein structures. Meanwhile, we show that the AlphaFold results need to be validated and should not be employed blindly, with the risk of obtaining an erroneous protein mechanism.

## Introduction

Over the past year, the artificial intelligence (AI)-based software AlphaFold created a buzz on the structural biology field. For the first time, a software was able to predict near-native protein folds from their genetic sequence (Jumper et al., 2021b). DeepMind’s AlphaFold transformed, in principle, the protein structure solving problem that has been around for the past 50 years into a trivial task. The number of research papers and preprints citing the method soared since its code was released in July 2021 (Callaway, 2022), with the accompanying article achieving about 1,000 citations (according to Google Scholar) in its first year.

The success of AlphaFold, and the analog RoseTTAFold approach (Baek et al., 2021) that appeared a few months later, is partially due to their open-source nature, which makes them readily and freely available to anyone who is interested in trying these software. Furthermore, by pairing it with the European Bioinformatics Institute (EBI), AlphaFold has taken structural biology to the next level, allowing big consortiums to perform protein structure prediction to entire genomes, including human, mouse, *Saccharomyces*, and *E. coli* (Tunyasuvunakool et al., 2021). The resulting structures were made available on a database maintained by the EBI, containing almost a million structures: https://alphafold.ebi.ac.uk.

The broad spread use of AI-based structure prediction leads us to ask the question: How reliable are the structures predicted by such models? Despite the growing number of success stories (Jumper et al., 2021a; Jumper et al., 2021b; Mosalaganti et al., 2021; Skolnick et al., 2021; Hartmann et al., 2022; Varadi et al., 2022), researchers are accumulating evidence showing that AI-based structure prediction methods are still not perfect (Perrakis and Sixma, 2021; Outeiral et al., 2022), and that there is ample room for improvement. In other words, some results suggest that both AlphaFold and RoseTTAFold are qualitatively great, but in many cases, they lack the level of details that is important to understand a protein function (Akdel et al., 2021; Eisenstein, 2021; Callaway, 2022).

High-resolution protein structures are also crucial for drug-discovery. The ability to readily access the structure of any protein of the human genome is very attractive to those developing new drug compounds. Using an AI-based tool to predict how drugs bind to these proteins is an even larger challenge that will probably not be overcome soon due to the limited publicly available data for small molecule binding (Mullard, 2021). In addition to that, AlphaFold lacks the precision to predict structural changes in consequence of mutations (Buel and Walters, 2022).

Working as a “computational microscope” molecular dynamics (MD) simulations are a unique tool to investigate biomolecules’ behavior with atomic resolution (Lee et al., 2009; Dror et al., 2012; Perilla et al., 2015). However, as most computational chemistry methods, the quality of MD results relies heavily, among other things, on the quality of the initial biomolecule structure (Bernardi and Pascutti, 2012; Vanommeslaeghe and MacKerell, 2015; Heo and Feig, 2018; Melo et al., 2018). If AI-based structure prediction software are able to predict protein folds to the atomic level, MD simulations should be able to profit from these structures and give similar results to those obtained with experimentally determined structures.

A particularly powerful way of using MD simulations is by using it hand-in-hand with experimental methods. Such form of use, among other things, allows computational biophysicists to overcome another limitation of MD simulations, namely the reliability of molecular mechanics force fields, particularly to treat ions and polarizable molecules (Neremberg and Head-Gordon, 2018; Yoo and Aksimentiev, 2018). In the past few years, taking advantage of steered MD protocols, our group has pioneered what we call *in silico* single-molecule force spectroscopy (*in silico* SMFS) (Bernardi et al., 2019; Sedlak et al., 2019; Sedlak et al., 2020). In this technique, steered MD (SMD) simulations are used in a wide-sampling approach to perform dozens to thousands of “pulling experiments,” in an analogy to what is done experimentally using atomic force microscopes (AFM). Allied to AFM-based SMFS, SMD has been successfully used to investigate a myriad of mechanically relevant biomolecular systems, including avidin:biotin (Grubmüller et al., 1996; Izrailev et al., 1997; Merkel et al., 1999), titin (Gao et al., 2002), human fibronectin (Gao et al., 2002), aquaporins (de Groot et al., 2009), among others.

The development of an *in silico* SMFS methodology, allowed us to go even further and to fine-tune mechanical properties of protein folds (Verdorfer et al., 2017). Besides protein design, our methodology allowed us to discover ultrastable protein complexes, and to decipher their intricate mechanostability mechanisms (Schoeler et al., 2014; Bernardi et al., 2019; Liu et al., 2020; Bauer et al., 2022). Among these ultrastable protein complexes, the ones formed by *Staphylococci* bacteria when adhering to humans are particularly interesting (Herman-Bausier and Dufrêne, 2018). These bacteria adhere to their hosts through proteins called adhesins (Dufrêne and Viljoen 2020). A particular class of adhesins, called microbial surface components recognizing adhesive matrix molecules (MSCRAMMs), play critical roles during infection, especially during the early step of adhesion when cells are exposed to mechanical stress. During the first steps of *Staphylococcus* infection, the interactions between these adhesins and proteins of the human extracellular matrix are a key virulence factor for these bacteria (Otto, 2008), and a crucial first step of biofilm formation (Latasa et al., 2006). These *Staphylococcus* biofilms are associated with more than half of all nosocomial infections (Jamal et al., 2018), with *Staphylococcus epidermidis* and *S. aureus* listed as the most common pathogens (Otto, 2008; Schilcher and Horswill, 2020).

To demonstrate the advantages and limitations of AI-based protein structure prediction methods, in this perspective article we used AlphaFold to predict the structures of several *S. aureus* MSCRAMM adhesins from the adhesion domain superfamily. First, a bioinformatics analysis was performed to select a set of adhesin sequences of different *S. aureus* strains that were then used as input for AlphaFold, when structural models were generated. Then, we employed our *in silico* SMFS methodology to characterize the mechanical properties of these proteins, comparing the results to those obtained with traditional structure biology methods.

## Application: Adhesin folding domains

### How good is AlphaFold to model full length adhesins?

After selecting 42 *S. aureus* adhesins from the adhesion superfamily, we used AlphaFold 2 through the VMD’s (Humphrey et al., 1996) QwikFold plugin (Gomes et al., 2022) batch mode to construct the models for full length apo adhesin protein models. Overall, AlphaFold 2 consistently predicted the canonical folds for N2 and N3 domains for all proteins and the homologous B repeats according to each protein domain organization (Foster and Hook, 1998; Ganesh et al., 2011; Foster et al., 2013) (Supplementary Figure S1; Supplementary Table S1). As expected, domains such as the serine aspartate or fibronectin binding repeats, as well as signal sequences, were predicted as disordered.

**FIGURE 1:**
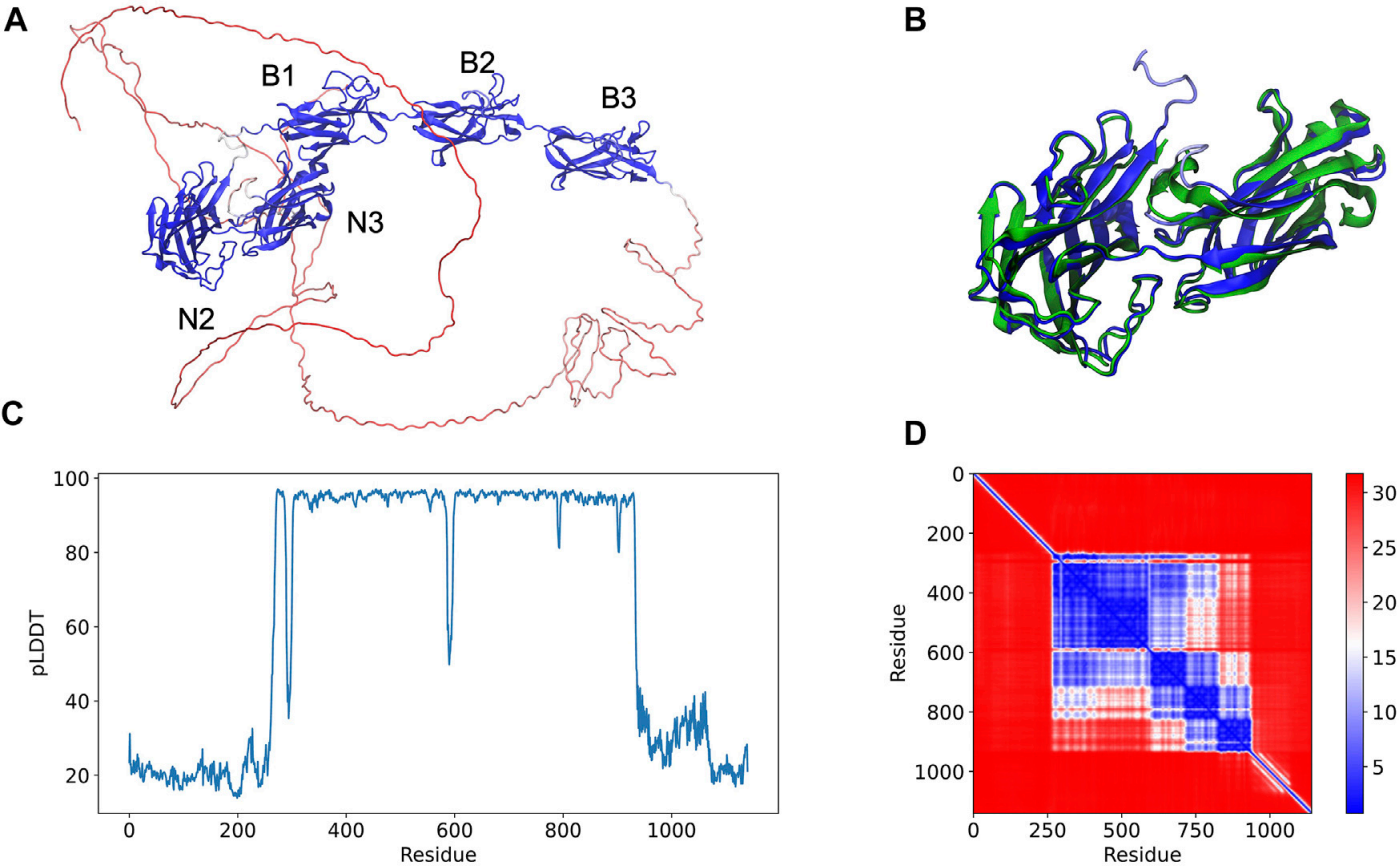
Full-length structure prediction of *S. aureus* serine-aspartate repeat protein (SdrE, Uniprot ID: Q932F7). **(A)** Top ranked SdrE model is represented in cartoon and its different domains are indicated. The protein is colored by the pLDDT scores generated by AlphaFold 2 where dark blue represent regions with very high quality (pLDDT > 90) and red represent regions with very low quality (pLDDT < 50). **(B)** Structural alignment between the N2 and N3 regions of the AlphaFold 2 model (dark blue) and SdrE crystallographic structure (cyan, PDB ID: 5WTA). **(C)** By residue pLDDT scores for the generated SdrE models. **(D)** Predicted alignment error (PAE) for the best ranked model. The color at (*x, y*) corresponds to the expected distance error in residue *x*’s position, when the prediction and true structure are aligned on residue *y*.

An example of an AlphaFold prediction for the serine-aspartate repeat-containing protein E (SdrE) is shown at Figure 1. The software predicted the Ig-like N2 and N3 domains in addition to B1, B2, and B3 repeats (Figure 1A). The N and C-terminal regions normally comprise disordered regions, such as peptide signals and the SD repeats, in the case of the serine aspartate repeat proteins (Figure 1A). A comparison between the available SdrE crystal structures in its unbound and bound states (PDB IDs 5wta and 5wtb, respectively) revealed a root mean square deviation (RMSD) of 1.71 and 2.79 Å, respectively, indicating that the model is a good approximation for the crystallographic structure of the Ig-like domains and can differentiate between bound and unbound states (Figure 1B). The major conformational change was found in the N2 domain: RMSD between SdrE in its unbound state (PDB ID: 5wta) and the model considering only N3 yields a RMSD of 0.84 Å.

The per-residue model quality can be evaluated by the predicted Local Distance Difference Test (pLDDT) quality scores, standard metric to evaluate AlphaFold generated models. In our studies, the pLDDT scores varied from ∼20 to 90 (Figure 1C) ranging from the disordered to folded regions of the proteins, which were predicted with high-quality. The confidence of the prediction can be accessed through the predicted alignment error (PAE) plots, which indicates the expected distance error in Angstroms (Figure 1D). PAE shows low error values for the N2, N3 (big blue square) and the B domains (three small squares), corroborating the pLDDT scores for the same region and indicating high confidence for the prediction of the mentioned domains.

### Is AlphaFold multimer reliable for *in-silico* force spectroscopy experiments?

**FIGURE 2:**
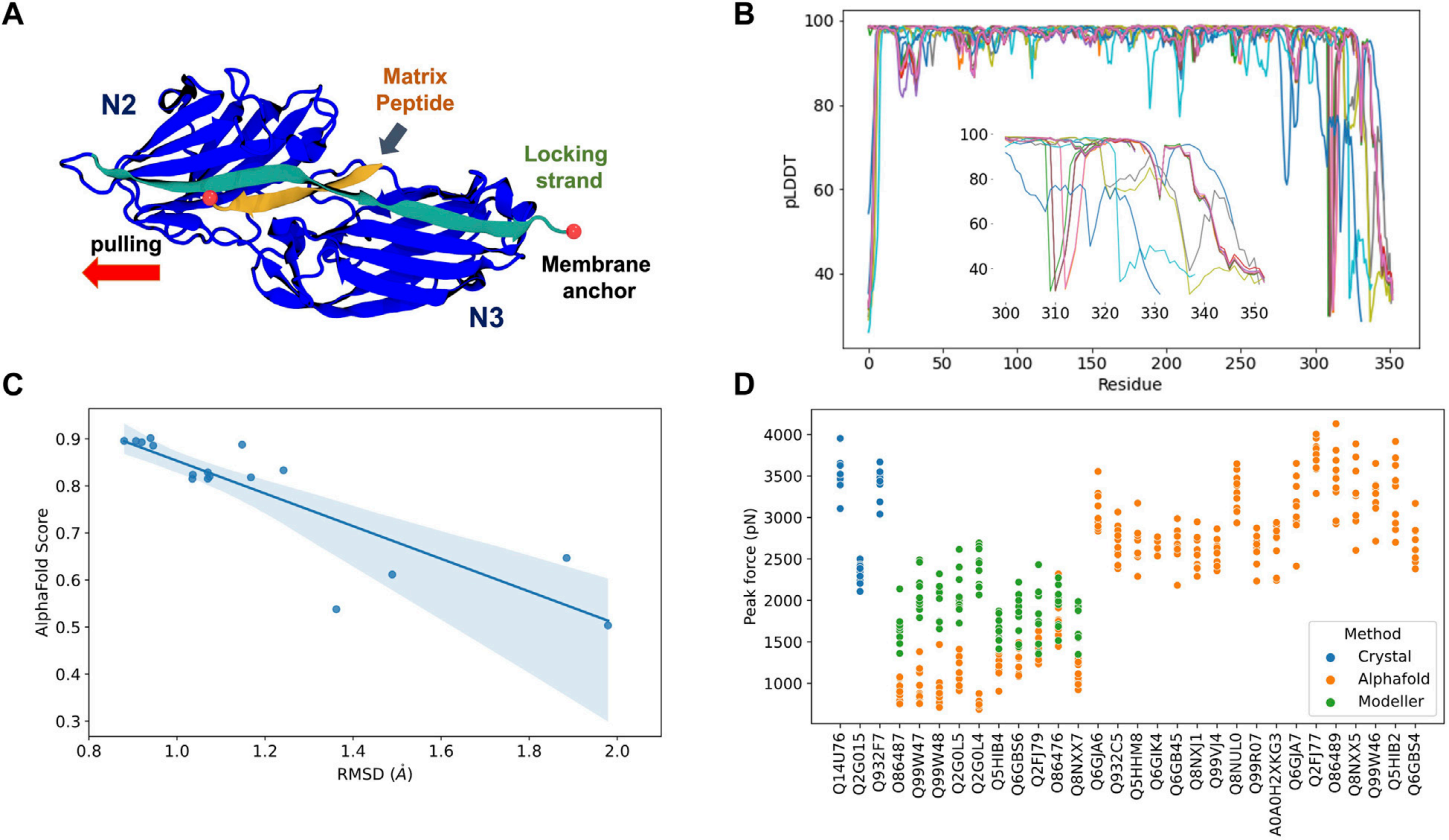
AlphaFold Multimer predictions for *S. aureus* adhesins. **(A)** Schematic view of adhesin’s Ig-like domain. Peptides from the host extracellular matrix are “locked” on a cleft between the N-terminal N2 and N3 domains, snugly accommodated by the “locking strand,” connecting N3 to N2 by *β*-Strand complementation (latch). SMD simulations were performed by keeping the C-terminal fixed as it would be anchored to the membrane while the peptide is pulled at the opposite direction by its N-terminal. **(B)** By residue pLDDT scores for the top ranked model at each complex prediction. The insert shows the variation among the C-terminal residues. **(C)** Comparison between AlphaFold Multimer score (ipTM) and RMSD values for equilibration pre-SMD simulations. **(D)** Peak Forces registered during SMD simulations for each studied complex. Color code indicates the origin of the departure structure: AlphaFold (orange), Modeller (green), or crystallographic (blue). Description of each accession entry are available at Supplementary Table S2.

Most *Staphylococcal* adhesins use a conserved “dock, lock, and latch” (DLL) mechanism—in which the host target, usually a peptide on the order of 15 residues, is first bound (dock), then buried (lock) between two immunoglobulin-like (Ig) fold domains N2 and N3 (Ponnuraj et al., 2003), and finally a “latch” connects N3 back to N2 holding the complex in place (Figure 2A). Small conformational changes in the Ig-like N2 and N3 domains could potentially impact force resilience when complexed to peptides if the DLL configuration is lost. Similar to the DLL mechanism, multiple biological phenomena rely on specific protein:protein interactions. Leveraging the initial protein structure prediction model, AlphaFold Multimer (Evans et al., 2022) was developed to predict structures of protein complexes for computational studies.

Here, we tested the reliability of *in silico* SMFS experiments performed with protein structures predicted by AlphaFold Multimer. To this end, we selected 27 adhesin sequences to be modelled in complex with extracellular matrix peptides (Supplementary Table S2). AlphaFold Multimer was used to construct models for the complexes through the QwikFold (Gomes et al., 2022) interface. Models were ranked by the interface predicted template modelling (ipTM) scores, used by AlphaFold Multimer, and the best ranked model for each complex was selected for SMD simulations. These simulations were carried out using NAMD 3.0 (Phillips et al., 2020), keeping the C-terminal of the adhesins anchored while the peptides were pulled at a constant speed. Such approach resembles an AFM-based SMFS experiments and can be used to measure the force upon which the complex dissociates (Gomes et al., 2022). Details and parameters are described at the Supplementary Material session. As control experiments, we also initiated SMD simulations using *S. aureus* crystallographic structures of three adhesin: peptide complexes: bone sialoprotein binding protein (BBP), clumping factor A (ClfA), and SdrE.

The predicted complexes were evaluated using pLDDT scores (Figure 2B). Most of the protein display high quality (pLDDT > 80), with exception of a very small portion of the N-terminal (10–15 residues) and a significant region of the C-terminal (last 50 residues, Figure 2B insert). The locking strand involved on the DLL mechanism is located on the C-terminal region of the protein structure, so this loss in model quality could impact the usability of the predicted structures in high-resolution experiments such as MD or SMD simulations.

A comparison of the RMSD calculated on an equilibration MD versus the general AlphaFold Multimer scores for the best ranked structures is shown at Figure 2C. We noticed that there is a correlation (Pearson correlation of 0.82, *p* < 0.005) between the model stability and the AlphaFold Multimer scores. Therefore, we can anticipate that high-scored structures present less deviation from its initial configuration, suggesting a more stable or resilient fold. AlphaFold Multimer scoring is based on an ipTM score that takes into account protein-protein interactions. This scoring function was shown to be more advantageous over the pTM and pLDDT scores used in AlphaFold 2 (Gao et al., 2022). The raking of the models is, in this case, a good indicator of model confidence based on the RMSD values.

After performing *in silico* SFMS experiments on all 27 complexes, we observed that the peak-force profiles ranged from ∼600 to 4,000 pN, a much broader range than previously simulated SdrE, BBP, and ClfA complexes, which were started from crystal structures (Figure 2D). *S. aureus* adhesins have been shown to be extremely mechanostable, with rupture forces consistently on the 2,000 pN regime (Milles et al., 2018). This force regime was also observed on this study maintaining the same *in silico* SFMS protocol used for all complexes. Considering the drastic difference in rupture forces, we found that the very low values (600–1,000 pN) seen for some of the complexes might have arisen from inaccurate initial structures. Visual inspection of the models with low rupture forces revealed that in most cases the locking strand was modelled in an unfavorable conformation to hold the peptide in the DLL configuration, which explains the observed behavior (Supplementary Figure S2).

To test this hypothesis, we re-modelled those complexes using comparative modelling with Modeller (Eswar et al., 2008) (Supplementary Table S2). The models were inspected for the presence of the locking strand and simulated according to the same protocol described above (peak force profiles are shown in Figure 2D). For all cases we recover the force resilience, with peaks reaching 2,000–3,000 pN range, confirming that a high-resolution initial structure is necessary to be used for MD and SMD simulations. It is important to note that, instead of Modeller, we could have employed custom templates in AlphaFold, which would have likely “forced” the structure into its correct conformation. However, the goal of this perspective article was to test how reliable “blind-runs” of AlphaFold are to predict structures to be used for *in silico* SMFS studies.

## Discussion

Protein structure prediction has been one of the grand challenges in Biology since the 1950’s (Dill et al., 2008; Dill and MacCallum, 2012). Several methods have been developed over the past 40 years that span from comparative modeling with the increase of experimentally determined protein structures by X-ray crystallography, nuclear magnetic resonance spectroscopy (NMR) and cryo-electron microscopy (cryo-EM) (Goh et al., 2016), but little progress was seen on *ab-initio* methodologies that rely solely on the protein sequence. But all of that changed upon the release of AlphaFold 2 in 2021. Although AlphaFold requires only the protein sequence as input, it should not be considered an *ab-initio* method since it is built on 50 years of knowledge of protein structure determination by experimental methods. AlphaFold tremendous success took advantage of both the recent explosion of AI methods, as well as the huge database of protein structure offered by the protein data bank (PDB) (Berman et al., 2000).

However, as nearly any other AI-based tool, AlphaFold is biased towards its training set, meaning that the search for unusual folds is unlikely to provide an accurate result. Despite the software’s success on the folded part of most proteins, AlphaFold lacks accuracy for regions where fewer sequences are available for alignment and intrinsically disordered regions, the latter are about one third of the human proteome, present in all proteomes of all kingdoms of life, and of all viral proteomes analyzed so far (Xue et al., 2012; Peng et al., 2015). It also struggles with protein interfaces in homo or hetero-multimers (Evans et al., 2022) and other aspects of protein structures such as co-factors, post-translational modifications and DNA or RNA complexes.

In order to show how revolutionary AlphaFold is for the single-molecule biophysics community, here we put AlphaFold to the test by using it to model full length *Staphylococcus* adhesins and estimate how stable are the protein structures. Ignoring the disordered regions, AlphaFold was able to model the Ig-like domains of MSCRAMMs adhesins as well as other key structural features of these proteins, such as the homologous B domains, for all the tested sequences. With a little refinement from *in-equilibrium* MD simulations, the generated structures could help to investigate the properties of many of the domains that still have an unknown function.

Additionally, we tested the newly developed AlphaFold Multimer to model adhesin:peptide complexes from different strains of *S. aureus* involved in biofilm formation. These protein complexes have been shown to be mechanically hyperstable, with a force resilience equivalent to that of a covalent bond at different pulling velocities (Milles et al., 2018), and lifetime under constant force that is in the order of hours (Huang et al., 2022). By comparing the force profile obtained from crystallographic structures of the complexes, we showed that AlphaFold Multimer failed to predict important key structural motifs for some of the protein complexes. Particularly, the locking strand of the adhesins, which are essential for interacting and locking the human target peptide in a tight complex with the N2 and N3 domains. However, it is still unclear why the predicted models worked for some cases and not for others. Limiting the set of templates to the ones where we know that the correct structures are present did not help to improve the results (data not shown). This highlights that its Multimer mode is not yet suitable to be blindly used as a peptide docking approach and the generated models should pass through a manual inspection to be suited for MD simulations.

In summary, AlphaFold 2 is a truly revolutionary tool that is bringing a new level of structural biology to bioinformatics. Although there are many areas where its methodology can be improved, the current algorithm can be clearly employed to work alongside single-molecule biophysics experiments. It is important to note that, as any other scientific tool, particularly new ones, AlphaFold 2 results cannot be employed blindly. Assessing the quality of the results and the usability of the predicted structures to infer function or mechanism to proteins is still the work of a trained scientist that can bring together data from multiple sources in a careful analysis of protein structure and dynamics.

## Data Availability

The original contributions presented in the study are included in the article/Supplementary Materials, further inquiries can be directed to the corresponding author.
