# Supplementary material for: Protein structure prediction in the era of AI: Challenges and limitations when applying to in silico force spectroscopy
Source: Front Bioinform. 2022 Oct 7;2:983306. doi: 10.3389/fbinf.2022.983306 (PMC9580946; doi:10.3389/fbinf.2022.983306)
Supplement: Supplementary file 1 [file DataSheet1.pdf]

Supplementary Material for:

Protein structure prediction in the era of AI: challenges and limitations when applying to *in silico* force spectroscopy

Priscila S. F. C. Gomes[1], Diego E. B. Gomes[1], Rafael C. Bernardi[1*]

[1]Department of Physics, College of Sciences and Mathematics, Auburn University, Auburn, AL, 36849

*** Correspondence:**
rcbernardi@auburn.edu

1 Supplementary figures

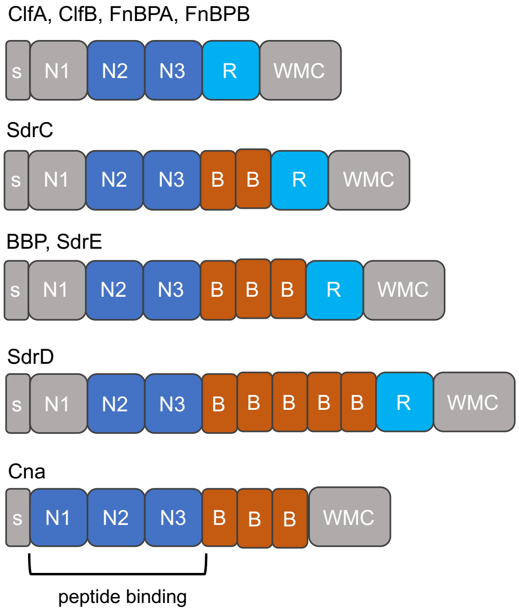

Supplementary Figure 1. *S. aureus* adhesin domain organization for clumping factor A (ClfA), Clumping factor B (ClfB), Fibronecting binding protein A (FnBPA), Fibronecting binding protein B (FnBPB), Serine-aspartate repeat protein C (SdrC), Serine-aspartate repeat protein D (SdrD), Serine-aspartate repeat protein E (SdrE), Bone sialoprotein binding protein (BBP) and Collagen binding adhesin (Cna). S, signal peptide; N-ter domains N1 to N3; B, homologous repeats; R, serine-aspartate or fibronectin binding repeats; W, wall-spanning region; M, membrane anchor; C, cytoplasmic tail. The ligand binding region is formed in a cleft between the N2 and N3 domains. Regions colored in gray had no defined structure predicted by AlphaFold 2. Peptide binding domain normally involves a gap between N2 and N3 regions, with exception of Cna, where it is located between N1 and N2 regions.

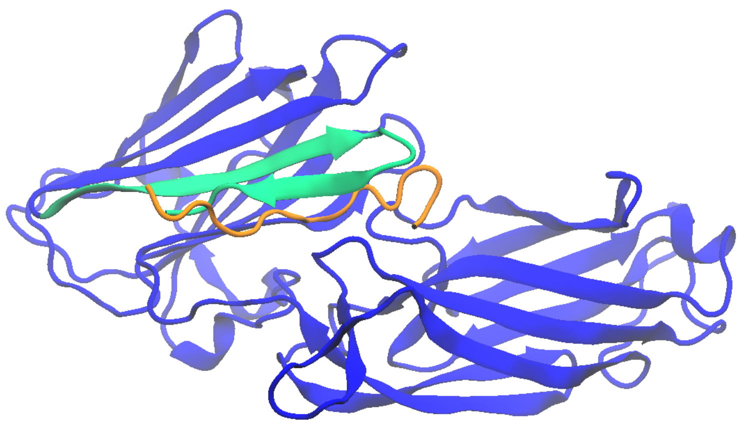

Supplementary Figure 2: AlphaFold Multimer model for SdrC (*S. aureus* strain Newman), colored in blue, complexed with fibrinopeptide alpha (Fgα), colored in orange. The locking strand is highlighted in green. We notice its different conformation from what is expected for the "dock, lock and latch" mechanism as illustrated in Figure 2A.

2 Supplementary Tables

Table S1: *S. aureus* adhesins modelled by AlphaFold 2. Full-length sequences were obtained from Uniprot according to the described entries. Predictions for N2/N3 are described by a "yes" and the number of modelled homologous B domains are also described.

Uniprot ID	Name	Organism	Length	N2/N3	B
Q6GJA6	Bone sialoprotein-binding protein	*Staphylococcus aureus* (strain MRSA252)	1137	y	3
Q53653	Clumping factor A	*Staphylococcus aureus* (strain Newman)	933	y	0
Q5HHM8	Clumping factor A	*Staphylococcus aureus* (strain COL)	933	y	0
Q6GB45	Clumping factor A	*Staphylococcus aureus* (strain MSSA476)	928	y	0
Q6GIK4	Clumping factor A	*Staphylococcus aureus* (strain MRSA252)	1029	y	0
Q8NXJ1	Clumping factor A	*Staphylococcus aureus* (strain MW2)	946	y	0
Q932C5	Clumping factor A	*Staphylococcus aureus* (strain Mu50 / ATCC 700699)	935	y	0
Q99VJ4	Clumping factor A	*Staphylococcus aureus* (strain N315)	989	y	0
Q2FUY2	Clumping factor B	*Staphylococcus aureus* (strain NCTC 8325 / PS 47)	877	y	0
Q5HCR7	Clumping factor B	*Staphylococcus aureus* (strain COL)	913	y	0

Q6G644	Clumping factor B	*Staphylococcus aureus* (strain MSSA476)	905	y	0
Q6GDH2	Clumping factor B	*Staphylococcus aureus* (strain MRSA252)	873	y	0
Q8NUL0	Clumping factor B	*Staphylococcus aureus* (strain MW2)	907	y	0
Q99R07	Clumping factor B	*Staphylococcus aureus* (strain Mu50 / ATCC 700699)	877	y	0
Q53654	Collagen adhesin	*Staphylococcus aureus*	1183	y	3
A7X6I5	Fibronectin-binding protein A	*Staphylococcus aureus* (strain Mu3 / ATCC 700698)	1038	y	0
P14738	Fibronectin-binding protein A	*Staphylococcus aureus* (strain NCTC 8325 / PS 47)	1018	y	0
Q2FE03	Fibronectin-binding protein A	*Staphylococcus aureus* (strain USA300)	1018	y	0
Q2YW62	Fibronectin-binding protein A	*Staphylococcus aureus* (strain bovine RF122 / ET3-1)	990	y	0
Q6GDU5	Fibronectin-binding protein A	*Staphylococcus aureus* (strain MRSA252)	965	y	0
Q8NUU7	Fibronectin-binding protein A	*Staphylococcus aureus* (strain MW2)	1015	y	0
A0A0H2XKG3	Fibronectin-binding protein B	*Staphylococcus aureus* (strain USA300)	940	y	0
O86487	Serine-aspartate repeat-containing protein C	*Staphylococcus aureus* (strain Newman)	947	y	2
Q2FJ79	Serine-aspartate repeat-containing protein C	*Staphylococcus aureus* (strain USA300)	947	y	2
Q2G0L5	Serine-aspartate repeat-containing protein C	*Staphylococcus aureus* (strain NCTC 8325 / PS 47)	995	y	2
Q5HIB4	Serine-aspartate repeat-containing protein C	*Staphylococcus aureus* (strain COL)	947	y	2
Q6GBS6	Serine-aspartate repeat-containing protein C	*Staphylococcus aureus* (strain MSSA476)	957	y	2
Q6GJA7	Serine-aspartate repeat-containing protein C	*Staphylococcus aureus* (strain MRSA252)	906	y	2
Q8NXX7	Serine-aspartate repeat-containing protein C	*Staphylococcus aureus* (strain MW2)	955	y	2
Q99W48	Serine-aspartate repeat-containing protein C	*Staphylococcus aureus* (strain Mu50 / ATCC 700699)	953	y	2
O86488	Serine-aspartate repeat-containing protein D	*Staphylococcus aureus* (strain Newman)	1315	y	5
Q2G0L4	Serine-aspartate repeat-containing protein D	*Staphylococcus aureus* (strain NCTC 8325 / PS 47)	1349	y	5
Q2FJ78	Serine-aspartate repeat-containing protein D	*Staphylococcus aureus* (strain USA300)	1381	y	5

Q6GBS5	Serine-aspartate repeat-containing protein D	*Staphylococcus aureus* (strain MSSA476)	1365	y	5
Q8NXX6	Serine-aspartate repeat-containing protein D	*Staphylococcus aureus* (strain MW2)	1347	y	5
Q99W47	Serine-aspartate repeat-containing protein D	*Staphylococcus aureus* (strain Mu50 / ATCC 700699)	1385	y	5
O86489	Serine-aspartate repeat-containing protein E	*Staphylococcus aureus* (strain Newman)	1166	y	3
Q2FJ77	Serine-aspartate repeat-containing protein E	*Staphylococcus aureus* (strain USA300)	1154	y	3
Q5HIB2	Serine-aspartate repeat-containing protein E	*Staphylococcus aureus* (strain COL)	1166	y	3
Q6GBS4	Serine-aspartate repeat-containing protein E	*Staphylococcus aureus* (strain MSSA476)	1141	y	3
Q932F7	Serine-aspartate repeat-containing protein E	*Staphylococcus aureus* (strain Mu50 / ATCC 700699)	1141	y	3
Q99W46	Serine-aspartate repeat-containing protein E	*Staphylococcus aureus* (strain N315)	1141	y	3

Table S2: *S. aureus* adhesins and the respective peptides complexes used on the AlphaFold Multimer predictions. Entries are organized by solving method; the ones marked as solved by Modeller were updated after obtention of low force profiles when using the predicted AlphaFold Multimer structures. The full-length sequences were obtained according to their Uniprot ID as described below and post-processed to contain only the N2-N3 domains as described at the Methods section below.

Solving method	Uniprot ID	Protein	ligand	Strain
X-ray (PDB ID:5CFA)	Q14U76	Bone sialoprotein-binding protein	Fibrinogen alpha	not informed
X-ray (PDB ID:2VR3)	Q2G015	Clumping factor A	Fibrinogen gamma	strain NCTC 8325 / PS 47
X-ray (PDB ID: 5WTB)	Q932F7	Serine-aspartate repeat-containing protein E	Complement factor H	Mu50 / ATCC 700699
AlphaFold	Q6GJA6	Bone sialoprotein-binding protein	Fibrinogen alpha	MRSA252
AlphaFold	Q932C5	Clumping factor A	Fibrinogen gamma	Mu50 / ATCC 700699
AlphaFold	Q5HHM8	Clumping factor A	Fibrinogen gamma	COL
AlphaFold	Q6GIK4	Clumping factor A	Fibrinogen gamma	MRSA252
AlphaFold	Q6GB45	Clumping factor A	Fibrinogen gamma	MSSA476
AlphaFold	Q8NXJ1	Clumping factor A	Fibrinogen gamma	MW2

AlphaFold	Q99VJ4	Clumping factor A	Fibrinogen gamma	N315
AlphaFold	Q8NUL0	Clumping factor B	Fibrinogen alpha	MW2
AlphaFold	Q99R07	Clumping factor B	Fibrinogen beta	Mu50 / ATCC 700699
AlphaFold	A0A0H2XKG3	Fibronectin-binding protein B	Fibrinogen beta	USA300
AlphaFold	Q6GJA7	Serine-aspartate repeat-containing protein C	Fibrinogen alpha	MRSA252
AlphaFold	Q2FJ77	Serine-aspartate repeat-containing protein E	Complement factor H	USA300
AlphaFold	O86489	Serine-aspartate repeat-containing protein E	Complement factor H	Newman
AlphaFold	Q8NXX5	Serine-aspartate repeat-containing protein E	Complement factor H	MW2
AlphaFold	Q99W46	Serine-aspartate repeat-containing protein E	Complement factor H	N315
AlphaFold	Q5HIB2	Serine-aspartate repeat-containing protein E	Complement factor H	COL
AlphaFold	Q6GBS4	Serine-aspartate repeat-containing protein E	Complement factor H	MSSA476
Modeller	Q2FUY2	Clumping factor B	Fibrinogen alpha	NCTC 8325 / PS 47
Modeller	Q2G0L5	Serine-aspartate repeat-containing protein C	Fibrinogen alpha	NCTC 8325 / PS 47
Modeller	Q99W48	Serine-aspartate repeat-containing protein C	Complement factor H	Mu50 / ATCC 700699
Modeller	Q6GBS6	Serine-aspartate repeat-containing protein C	Fibrinogen alpha	MSSA476
Modeller	Q2FJ79	Serine-aspartate repeat-containing protein C	Fibrinogen alpha	USA300
Modeller	Q8NXX7	Serine-aspartate repeat-containing protein C	Fibrinogen alpha	MW2
Modeller	Q5HIB4	Serine-aspartate repeat-containing protein C	Fibrinogen alpha	COL
Modeller	O86487	Serine-aspartate repeat-containing protein C	Fibrinogen alpha	Newman

Modeller	Q2G0L4	Serine-aspartate repeat-containing protein D	Fibrinogen alpha	NCTC 8325 / PS 47
Modeller	Q99W47	Serine-aspartate repeat-containing protein D	Complement factor H	Mu50 / ATCC 700699

3 Methods

3.1 Protein structure prediction and processing

We selected 54 *S. aureus* adhesins from the adhesion superfamily (InterPro: IPR008966) to have their full-length sequence (~1k residues) modelled. After eliminating redundancy, 42 sequences were retrieved from the Uniprot database according to the accession numbers described on Table S1. We used AlphaFold 2 (Jumper et al., 2021) through the VMD QwikFold plugin batch mode (Gomes et al., 2022) to construct the models for the full length apo proteins. Current implementation of AlphaFold 2 can generate up to 24 predictions for each sequence. All models were ranked by the predicted Local Distance Difference Test (pLDDT) quality scores.

From the same protein family, we selected 27 *S. aureus* adhesins to be modelled in complex with peptides from the human extracellular matrix (Table S2). The full-length sequences retrieved from Uniprot. Before structure prediction, the sequences were trimmed to contain only the N2 and N3 domains, necessary to dock and lock the peptides. To do that, all sequences were aligned using MAFFT (Nakamura et al., 2018). HMMER (Eddy, 2011) was used to generate a hidden-markov model profile using 3 *S. aureus* adhesins crystal structures that contained the Ig-like domains N2 and N3: bone sialoprotein binding protein, clumping factor B and serine-aspartate repeat-containing protein E (PDB IDs: 5CFA, 4F1Z, 5WTA, respectively). This profile was used to search against the aligned sequences and select the corresponding regions. Sequences for fibrinopeptides alfa, beta, gamma, in addition to complement factor H peptide were retrieved from available crystal structures for bone-sialoprotein binding protein (BBP), serine-aspartate repeat-containing protein G (SdrG from *S. epidermidis*), clumping factor A (ClfA) and serine-aspartate repeat-containing protein E (SdrE) (PDB IDs: 5CFA, 1R17, 2VR3, 5WTB, respectively). Proteins were later paired with each peptide based on information available on Uniprot. We used AlphaFold Multimer (Evans et al., 2022) through the QwikFold batch mode to construct the models. Five predictions were generated for each protein complex and ranked by the predicted interface template modelling (ipTM) scores, used by AlphaFold Multimer. The best ranked model for each complex was selected for steered molecular dynamics simulations.

Some adhesin:peptide complexes displayed very low force profiles upon SMD simulations. These were remodeled using Modeller (Eswar et al., 2008) using as templates BBP or SdrE crystal structures. Models were generated using standard parameters and the structures followed the same protocol described at the next session.

3.2 Steered molecular dynamics (SMD) simulations

SMD simulations were conducted using NAMD 3 (Phillips et al., 2020). All systems were prepared using the VMD QwikMD interface (Humphrey et al., 1996; Ribeiro et al., 2016) where the proteins

were solvated with TIP3 water model (Jorgensen and Jenson, 1998) and the total charge neutralized using NaCl 0.15 mol/L ion concentration. The CHARMM36 (Best et al., 2012) force field was used to describe the system and the simulations were performed under periodic boundary conditions in the NpT ensemble with temperature maintained at 300 K using Langevin dynamics for temperature and pressure coupling, the latter kept at 1 bar. A distance cut-off of 11.0 Å was applied to short-range non-bonded interactions, whereas long-range electrostatic interactions were treated using the particle-mesh Ewald (PME) (Darden et al., 1993) method. Before the SMD simulations all the systems were submitted to an energy minimization protocol for 1,000 steps. Additionally, an MD simulation with position restraints in the protein backbone atoms was performed for 1 ns, with temperature ramping from 0k to 300 K in the first 0.5 ns, which served to pre-equilibrate the system. For SMD, adhesins were C-terminal anchored, while peptides were pulled at a constant speed of 5x10-5 Å/ps with a 5 kcal/mol/Å^2 spring constant for 10 ns. Production runs were generated in ten replicas for each complex. We also selected 3 *S. aureus* crystallographic structures of adhesin:peptide complexes: BBP, ClfA and SdrE (PDB IDs: 5CFA, 2VR3 and 5WTB) to be simulated as control. Root mean square deviation (RMSD) values were calculated for the equilibrium simulation pre-SMD, for all systems. Protein images were generated using VMD; plots were rendered using python scripts.

4 References

Best, R. B., Zhu, X., Shim, J., Lopes, P. E. M., Mittal, J., Feig, M., et al. (2012). Optimization of the additive CHARMM all-atom protein force field targeting improved sampling of the backbone φ, ψ and side-chain χ1 and χ2 Dihedral Angles. *Journal of Chemical Theory and Computation* 8, 3257–3273. doi: 10.1021/CT300400X.

Darden, T., York, D., and Pedersen, L. (1993). Particle mesh Ewald: An N·log(N) method for Ewald sums in large systems. *The Journal of Chemical Physics* 98, 10089. doi: 10.1063/1.464397.

Eddy, S. R. (2011). Accelerated Profile HMM Searches. *PLOS Computational Biology* 7, e1002195. doi: 10.1371/JOURNAL.PCBI.1002195.

Eswar, N., Eramian, D., Webb, B., Shen, M.-Y., and Sali, A. (2008). Protein structure modeling with MODELLER. *Methods Mol Biol* 426, 145–59. doi: 10.1007/978-1-60327-058-8_8.

Evans, R., O'Neill, M., Pritzel, A., Antropova, N., Senior, A., Green, T., et al. (2022). Protein complex prediction with AlphaFold-Multimer. *bioRxiv*, 2021.10.04.463034. doi: 10.1101/2021.10.04.463034.

Gomes, D. E. B., da Silva Figueiredo Celestino Gomes, P., and C Bernardi, R. (2022). QwikMD 2.0: bridging the gap between sequence, structure, and protein function. *Biophysical Journal* 121, 132a.

Humphrey, W., Dalke, A., and Schulten, K. (1996). VMD: Visual molecular dynamics. *Journal of Molecular Graphics* 14, 33–38. doi: 10.1016/0263-7855(96)00018-5.

Jorgensen, W. L., and Jenson, C. (1998). Temperature dependence of TIP3P, SPC, and TIP4P water from NPT Monte Carlo simulations: Seeking temperatures of maximum density. *Journal of Computational Chemistry* 19, 1179–1186. doi: 10.1002/(SICI)1096-987X(19980730)19:10<1179::AID-JCC6>3.0.CO;2-J.

Jumper, J., Evans, R., Pritzel, A., Green, T., Figurnov, M., Ronneberger, O., et al. (2021). Highly accurate protein structure prediction with AlphaFold. *Nature 2021 596:7873* 596, 583–589. doi: 10.1038/s41586-021-03819-2.

Nakamura, T., Yamada, K. D., Tomii, K., and Katoh, K. (2018). Parallelization of MAFFT for large-scale multiple sequence alignments. *Bioinformatics* 34, 2490–2492. doi: 10.1093/bioinformatics/bty121.

Phillips, J. C., Hardy, D. J., Maia, J. D. C., Stone, J. E., Ribeiro, J. v., Bernardi, R. C., et al. (2020). Scalable molecular dynamics on CPU and GPU architectures with NAMD. *The Journal of Chemical Physics* 153, 044130. doi: 10.1063/5.0014475.

Ribeiro, J. v., Bernardi, R. C., Rudack, T., Stone, J. E., Phillips, J. C., Freddolino, P. L., et al. (2016). QwikMD — Integrative Molecular Dynamics Toolkit for Novices and Experts. *Scientific Reports 2016 6:1* 6, 1–14. doi: 10.1038/srep26536.
